# Influence of Rugate Filters on the Spectral Manifestation of Tamm Plasmon Polaritons

**DOI:** 10.3390/ma14051282

**Published:** 2021-03-08

**Authors:** Victor Yu. Reshetnyak, Igor P. Pinkevych, Timothy J. Bunning, Dean R. Evans

**Affiliations:** 1 Physics Faculty, Taras Shevchenko National University of Kyiv, 01601 Kyiv, Ukraine; VReshetnyak@univ.kiev.ua; 2 Air Force Research Laboratory, Materials and Manufacturing Directorate, Wright-Patterson Air Force Base, OH 45433, USA

**Keywords:** Tamm plasmon, Bragg mirror, rugate filter, band gap, light reflection and transmission

## Abstract

This study theoretically investigated light reflection and transmission in a system composed of a thin metal layer (Ag) adjacent to a rugate filter (RF) having a harmonic refractive index profile. Narrow dips in reflectance and peaks in transmittance in the RF band gap were obtained due to the excitation of a Tamm plasmon polariton (TPP) at the Ag–RF interface. It is shown that the spectral position and magnitude of the TPP dips/peaks in the RF band gap depend on the harmonic profile parameters of the RF refractive index, the metal layer thickness, and the external medium refractive index. The obtained dependences for reflectance and transmittance allow selecting parameters of the system which can be optimized for various applications.

## 1. Introduction

Recently, much attention has been paid to the study of Tamm plasmons and their applications. Tamm plasmon (otherwise known as Tamm plasmon polariton—TPP) is an electromagnetic mode localized at the interface between a metal film and a dielectric Bragg mirror [1,2,3,4,5]. Kaliteevsky et al. [3] called these localized modes Tamm plasmons by analogy with the electron states localized near the surface of a solid crystal and predicted by Tamm [6]. TPP localization is provided by the negative dielectric constant of the metal on the one side and the photonic stop band of the Bragg mirror on the other. In contrast to ordinary surface plasmon polaritons that are only transverse magnetic (TM) -polarized and have the dispersion relations outside the light cone, TPPs can be transverse electric (TE)- and TM-polarized with the dispersion inside the light cone. This allows direct optical excitation of TPP with TE- and TM-polarized light at any angle of incidence without the need for a prism or grating [3,4,5,6,7]. These unique capabilities make TPPs very attractive for various photoelectronic applications.

TPPs manifest themselves optically in the form of narrow dips/peaks in the reflection/transmission spectra in the spectral region corresponding to the photonic band gap of the Bragg mirror. TPPs are a good alternative to conventional surface plasmons, with potential applications for sensors [8,9,10,11,12,13,14], Tamm plasmon-based lasers [15,16,17], optical switches and filters [18,19,20], liquid crystal-tuned Tamm plasmon devices [21,22,23], and selective thermal and light emitters [24,25,26,27,28].

Over the past couple of decades, the optical properties of rugate filters (RFs) have been intensively studied [29]. RFs are dielectric thin films with a smooth periodic profile of the refractive index, giving rise to spectral band gaps like Bragg mirrors, typically composed of a square wave profile of the refractive index. The smooth profile of the RF refractive index makes it possible to improve many characteristics of optical devices compared to dielectric multilayer Bragg mirrors. RFs provide a photonic band gap without significant ripples in the reflection spectrum outside the band gap and without its higher harmonics, and they enable the possibility to overlay multiple harmonic waves (giving rise to multiple spectral notches). Furthermore, RFs have substantially higher laser-induced damage thresholds with respect to Bragg mirrors (see, for example, [29,30,31,32]). There have been numerous methods developed for obtaining RFs [33,34,35,36,37]; for example, porous silicon-based RFs represent a popular approach [31,38,39,40,41,42].

In this paper, RFs are explored as structures to excite Tamm plasmons, replacing the previously studied multilayer Bragg mirrors. The influence of RF parameters on the TPP excitation at the metal–RF interface is theoretically studied. The paper is organized as follows. Section 2 introduces a model of the metal–RF structure and derives equations allowing for the calculation of reflectance and transmittance of this structure in the RF band gap. Results of numerical calculations for a system using an Ag layer and their discussion are presented in Section 3. Section 4 presents some brief conclusions.

## 2. Theoretical Model and Basic Equations

**Figure 1:**
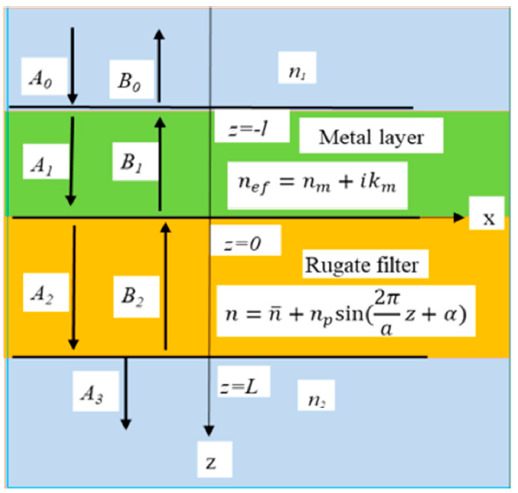
Schematic of the “metal layer–rugate filter (RF)” structure together with directions of the light beams propagating in the system and the refractive indices of the constituent substances.

Consider a structure, composed of an RF with a periodic dielectric function along the *z*-axis and a metal layer adjacent to the RF. A light beam, polarized along the *x*-axis, is normally incident on the metal layer along the *z*-axis and propagates through the metal and adjacent dielectric RF. Assuming that the principal axes of the RF dielectric tensor coincide with the Cartesian axes, the Cartesian indices of the electric and magnetic vectors can be omitted. A schematic of the structure together with directions of the light beams propagating in the system is presented in Figure 1.

In the area above the metal layer, z≤−l, the electromagnetic field of the incident and reflected beams is described by the electric and magnetic vectors,

(1) $$\begin{array}{l} E_{0}(z)=A_{0}\exp(ikn_{1}z)+B_{0}\exp(-ikn_{1}z),\\ H_{0}(z)=\frac{n_{1}}{\mu_{0}c}\left[A_{0}\exp(ikn_{1}z)-B_{0}\exp(-ikn_{1}z)\right] \end{array}$$

where k=ω/c, $n_{1}$ is a refractive index of the medium in front of the metal layer, and $A_{0}$, $B_{0}$ are the amplitudes of the incident and reflected beams, respectively.

In the metal layer, −l≤z≤0, the electric and magnetic vectors of the wave field are written as

(2) $$\begin{array}{l} E_{1}(z)=A_{1}\exp(ikn_{ef}z)+B_{1}\exp(-ikn_{ef}z),\\ H_{1}(z)=\frac{n_{ef}}{\mu_{0}c}[A_{1}\exp(ikn_{ef}z)-B_{1}\exp(-ikn_{ef}z)] \end{array}$$

where $n_{ef}=n_{m}+ik_{m}$ is the complex refractive index of the metal, and $A_{1}$, $B_{1}$ are the amplitudes of the forward and backward waves, respectively.

In the RF area, 0≤z≤L, the dielectric tensor is periodic along the *z*-axis. Designating the period with the letter a, the dielectric tensor principal value $\varepsilon_{xx}\left(z\right)$ presented in the wave equation can be expanded in a Fourier series,

(3) $$\varepsilon_{xx}(z)=\varepsilon_{0}+\sum_{\begin{array}{l} m=-\infty \\ \left(m\neq 0\right) \end{array}}^{\infty }\varepsilon_{m}e^{i\frac{2\pi }{a}mz}$$

For the sake of simplicity, neglecting absorption in the RF, $\varepsilon_{0}={\bar{n}}^{2}$ can be used in Equation (3), where $\bar{n}$ is the RF average refractive index. Next, consider the case when a wavelength of the incident beam is close to the Bragg wavelength $\lambda_{r}$ satisfying the Bragg resonance condition $\frac{\lambda_{r}}{\bar{n}}m=2a$, where m is an integer. Then, solving the wave equation in the RF area, one can use the coupled wave method [43,44] and present the electric vector of the electromagnetic field in the form of a superposition of the forward and backward waves,

(4) $$E_{2}(z)=A_{2}(z)\exp(ik\bar{n}z)+B_{2}(z)\exp(-ik\bar{n}z)$$

where $A_{2}(z)$ and $B_{2}(z)$ are the slowly varying functions satisfying the Kogelnik Equations [39],

(5) $$\begin{array}{l} \frac{\partial A_{2}(z)}{\partial z}=i\chi_{m}\exp\left(-i2\delta z\right)B_{2}(z),\\ \frac{\partial B_{2}(z)}{\partial z}=-i\chi_{-m}\exp\left(i2\delta z\right)A_{2}(z) \end{array}$$

In Equation (5) $\delta =k\bar{n}-\frac{\pi }{a}m$ is the offset from the Bragg resonance, $\chi_{\pm m}=k\varepsilon_{\pm m}/2\bar{n}$. Solving Equation (5) under the assumptions,

(6) $$\left|\delta \right|a<<1,\;\left|\chi_{\pm m}\right|a<<1$$

one can obtain solutions in the following form [45]:

(7) $$\begin{array}{l} A_{2}(z)=\left[a_{1}\exp(-\gamma_{m}z)+a_{2}\exp(\gamma_{m}z)\right]\exp\left(-i\delta z\right),\\ B_{2}(z)=\left[a_{1}r_{m}\exp(-\gamma_{m}z)+a_{2}\frac{\chi_{-m}}{\chi_{m}}\frac{1}{r_{m}}\exp(\gamma_{m}z)\right]\exp\left(i\delta z\right) \end{array}$$

where $\gamma_{m}\approx {\left(\chi_{m}\chi_{-m}-\delta^{2}\right)}^{1/2}$, $r_{m}=\frac{i\chi_{-m}}{\gamma -i\delta }$, and coefficients $a_{1},a_{2}$ can be determined from the boundary conditions.

Using the Maxwell equation $rot\vec{E}=-\partial \vec{B}/\partial t$ and Equations (4) and (5), the following expression is obtained for the magnetic field vector in the RF area:

(8) $$\begin{array}{l} H_{2}(z)=-\frac{1}{\mu_{0}c}\left[\frac{\chi_{-m}}{k}\exp[-i(k\bar{n}-2\delta )z]-\bar{n}\exp(ik\bar{n}z)\right]A_{2}(z)+\\ +\frac{1}{\mu_{0}c}\left[\frac{\chi_{m}}{k}\exp[i(k\bar{n}-2\delta )z]-\bar{n}\exp(-ik\bar{n}z)\right]B_{2}(z) \end{array}$$

In the area below RF, z≥L, there is only an outgoing wave described by the electric and magnetic vectors

(9) $$E_{3}(z)=A_{3}\exp(ikn_{2}z),H_{3}\left(z\right)=\frac{n_{2}}{\mu_{0}c}A_{3}\exp(ikn_{2}z)$$

where $n_{2}$ is a refractive index of the medium in the area z≥L.

Now, to get expressions for the amplitudes of the reflected, $B_{0}$, and transmitted, $A_{3}$, waves, one can write down the boundary conditions for the electric and magnetic vectors at z=−l, z=0 and z=L:

(10) $$\begin{array}{l} A_{0}\exp(-ikn_{1}l)+B_{0}\exp(ikn_{1}l)=A_{1}\exp(-ikn_{ef}l)+B_{1}\exp(ikn_{ef}l),\\ A_{0}\exp(-ikn_{1}l)-B_{0}\exp(ikn_{1}l)=\frac{n_{ef}}{n_{1}}[A_{1}\exp(-ikn_{ef}l)-B_{1}\exp(ikn_{ef}l)] \end{array}$$

(11) $$\begin{array}{l} A_{1}+B_{1}=A_{2}(0)+B_{2}(0),\\ n_{ef}\left(A_{1}-B_{1}\right)=-\left(\frac{\chi_{-m}}{k}-\bar{n}\right)A_{2}(0)+\left(\frac{\chi_{m}}{k}-\bar{n}\right)B_{2}(0) \end{array}$$

(12) $$\begin{array}{l} A_{2}(L)\exp(ik\bar{n}L)+B_{2}(L)\exp(-ik\bar{n}L)=A_{3}\exp(ikn_{2}L),\\ \left(-\frac{\chi_{-m}}{k}+\bar{n}\right)A_{2}(L)\exp(i\delta L)+\left(\frac{\chi_{m}}{k}-\bar{n}\right)B_{2}(L)\exp(-i\delta L)=(-1)m^{p}n_{2}A_{3}\exp(ikn_{2}L) \end{array}$$

when writing Equation (12), the conditions $\left(k\bar{n}-\delta \right)L=m\pi L/a$ and L/a=p are used, where p is the number of periods of dielectric function along the RF length.

Substituting Equation (7) into Equations (10)–(12), these equations can be solved, and expressions for the reflectance, $R={\left|B_{0}/A_{0}\right|}^{2}$, and transmittance, $T=\left(n_{2}/n_{1}\right){\left|A_{3}/A_{0}\right|}^{2}$, of the system can be obtained in the following form:

(13) $$R={\left|1-\frac{\frac{2n_{ef}}{n_{1}}[1-C+\exp(-i2kn_{ef}l)]}{\left(1-\frac{n_{ef}}{n_{1}})(C-1)+(1+\frac{n_{ef}}{n_{1}})\exp(-i2kn_{ef}l\right)}\right|}^{2}$$

(14) $$\begin{array}{l} T=16\frac{n_{2}}{n_{1}}{\left|\frac{(1+r_{m})\left(c_{3}+\frac{\chi_{-m}}{\chi_{m}}\frac{1}{r_{m}}c_{4}\right)-(1+\frac{\chi_{-m}}{\chi_{m}}\frac{1}{r_{m}})\left(c_{3}+r_{m}c_{4}\right)}{\left(c_{1}+r_{m}c_{2}\right)\left(c_{3}+\frac{\chi_{-m}}{\chi_{m}}\frac{1}{r_{m}}c_{4}\right)\exp(\gamma_{m}L)-\left(c_{1}+\frac{\chi_{-m}}{\chi_{m}}\frac{1}{r_{m}}c_{2}\right)\left(c_{3}+r_{m}c_{4}\right)\exp(-\gamma_{m}L)}\right|}^{2}\times \\ {\left|\frac{1}{\left(1-\frac{n_{ef}}{n_{1}})(C-1)\exp(ikn_{ef}l)+(1+\frac{n_{ef}}{n_{1}})\exp(-ikn_{ef}l\right)}\right|}^{2} \end{array}$$

where

(15) $$C=2\frac{\left(1+r_{m})\left(c_{3}+\frac{\chi_{-m}}{\chi_{m}}\frac{1}{r_{m}}c_{4}\right)\exp(\gamma_{m}L)-(1+\frac{\chi_{-m}}{\chi_{m}}\frac{1}{r_{m}})\left(c_{3}+r_{m}c_{4}\right)\exp(-\gamma_{m}L\right)}{\left(c_{1}+r_{m}c_{2}\right)\left(c_{3}+\frac{\chi_{-m}}{\chi_{m}}\frac{1}{r_{m}}c_{4}\right)\exp(\gamma_{m}L)-\left(c_{1}+\frac{\chi_{-m}}{\chi_{m}}\frac{1}{r_{m}}c_{2}\right)\left(c_{3}+r_{m}c_{4}\right)\exp(-\gamma_{m}L)}$$

(16) $$\begin{array}{l} c_{1}=1+\frac{\bar{n}}{n_{ef}}-\frac{\chi_{-m}}{k}\frac{1}{n_{ef}},\;c_{2}=1-\frac{\bar{n}}{n_{ef}}+\frac{\chi_{m}}{k}\frac{1}{n_{ef}},\\ c_{3}=(-1){}^{mp}\left(\bar{n}-n_{2}-\frac{\chi_{-m}}{k}\right),\;c_{4}=(-1){}^{mp}\left(-\bar{n}-n_{2}+\frac{\chi_{m}}{k}\right) \end{array}$$

To consider the case when the RF refractive index is a periodic function, the following form is used:

(17) $$n\left(z\right)=\bar{n}+n_{p}\sin\left(\frac{2\pi }{a}z+\alpha \right)$$

In Equation (17), varying α changes the value of the refractive index in the locations immediately adjacent to the metal; this gives a more general form of the harmonic function of the refractive index. Assuming $n_{p}<<\bar{n}$, the corresponding dielectric function is $\varepsilon_{xx}(z)\approx {\bar{n}}^{2}+2n_{p}\bar{n}\sin\left(\frac{2\pi }{a}z+\alpha \right)$**.** According to Equation (3), it has the following non-zero Fourier components: $\varepsilon_{0}={\bar{n}}^{2},\;\varepsilon_{1}=-in_{p}\bar{n}e^{i\alpha },\;\varepsilon_{-1}=in_{p}\bar{n}e^{-i\alpha }$**.** In this case, in Equations (13)–(16), the integer m must be set to 1; therefore, the Bragg wavelength $\lambda_{r}=2\bar{n}a$.

## 3. Results of Numerical Calculations and Discussion

For numerical calculations, we take the “Ag layer–RF” structure with parameters that are close to those previously studied for a system consisting of an Ag plate and a Bragg mirror composed of alternating TiO_2_ and SiO_2_ layers with a thickness of $d_{1}=50.4\;\mathrm{nm}$ and $d_{2}=86.7\;\mathrm{nm}$, respectively. In this system, the Tamm plasmon resonances were experimentally detected in the visible region [46]; therefore, we take the same period for the RF refractive index, $a=d_{1}+d_{2}=137.1$ nm, and average refractive index $\bar{n}=1.85$, calculated as $\bar{n}=\left(n_{TiO_{2}}d_{1}+n_{SiO_{2}}d_{2}\right)/\left(d_{1}+d_{2}\right)$ where $n_{TiO_{2}}$ and $n_{SiO_{2}}$ are the refractive indices of TiO_2_ and SiO_2_, respectively [47,48]. 

The conditions in Equation (6) impose restrictions on the maximum values of a magnitude $n_{p}$ of the RF refractive index modulation. Indeed, the RF band gap is proportional to $\chi_{\pm 1}$ [45] and, therefore, to $n_{p}$ (taking into account that $\left|\chi_{\pm 1}\right|=\left|k\varepsilon_{\pm 1}/2\bar{n}\right|\approx \pi n_{p}/\lambda_{r}$). The value $n_{p}=0.2$ or values close to it ensure the fulfillment of the conditions in Equation (6) and are used further in the numerical calculations. For the complex refractive index of Ag, frequency dispersion is taken into account [49]. The refractive indices of media before the Ag layer and after the RF film are parameters that can be varied.

We calculated the reflectance and transmittance spectra of the system composed of the RF film with a refractive index profile described by Equation (17) and the Ag layer placed at the top of the RF (see Figure 1). For calculations, we set the refractive indices of the media before the Ag layer and after the RF as $n_{1}=n_{2}=1$, the Ag layer thickness as 45 nm, and the RF thickness as *L* = 1919.4 nm (i.e., 14 periods of the RF refractive index). Values of α in Equation (17) used for the calculations are presented in Table 1 and selected only as an example.

**Figure 2:**
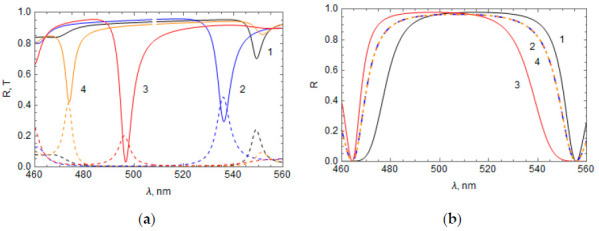
(**a**) Reflectance and transmittance spectra of the “Ag layer–RF” system at different initial phase *α*: α=−π/2 (1, black), 0 (2, blue), π/2 (3, red), and π (4, orange); reflectance—Solid lines, transmittance—Dashed lines, Ag film thickness = 45 nm. (**b**) Reflectance spectrum of only the RF, where the numbers near the curves correspond with the α values used in Figure 2a.

For each initial (at *z* = 0) phase α of the RF refractive index, reflection dips and transmission peaks in the spectral region of the RF band gap were obtained, associated with the excitation of TPP at the Ag layer–RF interface. Results of calculations are shown in Figure 2a for all α values presented in Table 1. Figure 2b shows the reflectance of only the RF with the same parameters that were used for each case of α in Figure 2a. As can be seen, the inclusion of the thin layer of metal on top of the RF thin film drastically modified the spectral content of the optical architecture.

It can be seen from Figure 2a that the position and magnitude of the TPP spectral bands depend strongly on the initial phase α, as the TPP wavelength decreases with increasing *α* (Figure 2b). Comparing the RF refractive index profiles, which correspond to bands 1 and 3 [$n\left(z\right)=\bar{n}\pm n_{p}\cos\left(2\pi z/a\right)$] or 2 and 4 $n\left(z\right)=\bar{n}\pm n_{p}\sin\left(2\pi z/a\right)$ we can conclude that the sign of the term added to the RF average refractive index, $\bar{n}$, significantly affects the TPP wavelength.

**Figure 3:**
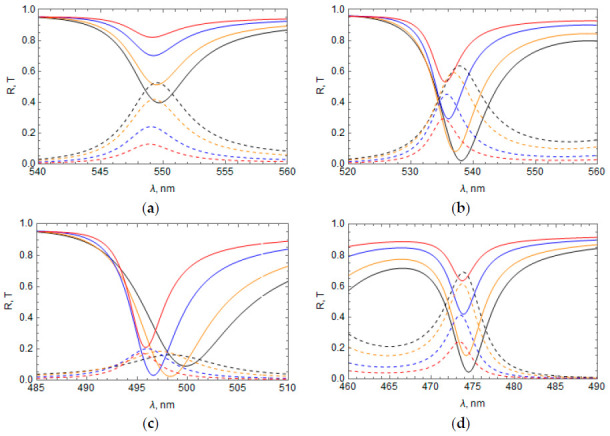
Influence of the Ag film thickness *l* on the reflectance and transmittance of the system for different RF profiles *n*(*z*); (**a**) TPP band 1, $n\left(z\right)=\bar{n}-n_{p}\cos\left(2\pi z/a\right)$; (**b**) TPP band 2, $n\left(z\right)=\bar{n}+n_{p}\sin\left(2\pi z/a\right)$; (**c**) TPP band 3, $n\left(z\right)=\bar{n}+n_{p}\cos\left(2\pi z/a\right)$; (**d**) TPP band 4, $n\left(z\right)=\bar{n}-n_{p}\sin\left(2\pi z/a\right)$. Reflectance—Solid lines, transmittance—Dashed lines, *l* = 30 nm—Black, 35 nm—Orange, 45 nm—Blue, 55 nm—Red. The TPP bands refer to those found in Figure 2.

The influence of the Ag layer thickness on the TPP wavelength and the TPP dip/peak magnitude is shown in Figure 3 for the bands 1 (Figure 3a), 2 (Figure 3b), 3 (Figure 3c), and 4 (Figure 3d). The TPP wavelength and reflectance dip, as a rule, decrease (the TPP transmittance peak increases) with an increase in Ag layer thickness; however, for peak 3, there is an optimal thickness of 35 nm.

**Figure 4:**
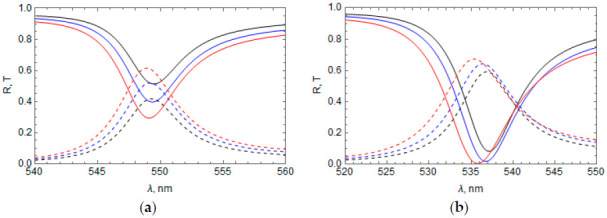

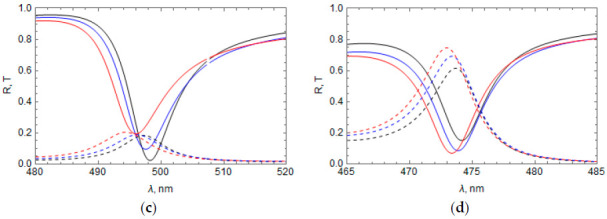
Influence of the refractive index of the medium above the Ag layer, *n*_1_, on the reflectance and transmittance: $n_{1}$ = 1 (black line), 1.5 (blue), and 2.5 (red); (**a**) TPP band 1; (**b**) TPP band 2; (**c**) TPP band 3; (**d**) TPP band 4. Reflectance—Solid lines, transmittance—Dashed lines, thickness of the Ag layer = 35 nm.

In Figure 4, we show the influence of the refractive index of the medium above the Ag layer, $n_{1}$, on the reflectance and transmittance of the system with the RF profiles n(z) from Table 1. In all of these cases, an increase in the refractive index of the medium above the Ag layer leads to a TPP wavelength decrease and a change in the dip/peak magnitude.

**Figure 5:**
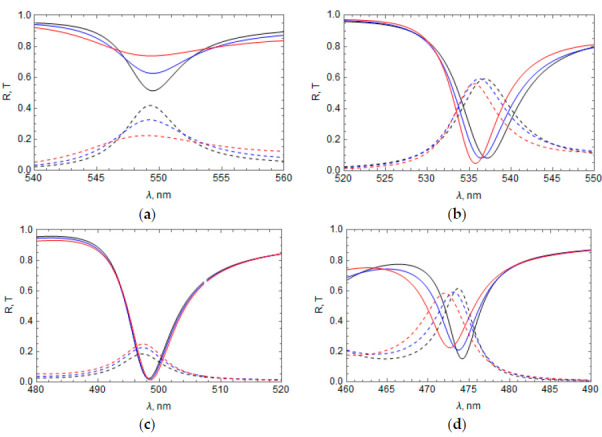
Influence of the refractive index of medium below the RF, *n*_2_ on reflectance and transmittance: $n_{2}$ = 1 (black line), 1.5 (blue line), and 2.5 (red line); (**a**) TPP band 1; (**b**) TPP band 2; (**c**) TPP band 3; (**d**) TPP band 4. Reflectance—Solid lines, transmittance—Dashed lines, thickness of the Ag layer = 35 nm.

The influence of the refractive index of the medium below the RF, $n_{2}$, on the reflectance and transmittance of the system is shown in Figure 5 for the same cases as in Figure 4. As in the case of a change of the refractive index of the medium above the Ag layer, an increase in the medium refractive index below the RF shifts the TPP bands toward shorter wavelengths and changes the dip/peak magnitude.

As seen from Figure 4 and Figure 5, the dependence of reflectance and transmittance on the refractive index of the media above the metal layer and below the RF is different for different profiles n(z) of the RF. Furthermore, the impact of $n_{2}$ becomes weaker when the transmittance of the system decreases. It takes place for some profiles n(z) (see, for example, Figure 4c) or with increasing the number of periods n(z) along the RF. In both of these cases, the electromagnetic field at the boundary of the RF with the adjacent medium (after the RF) becomes weaker; therefore, the influence of the adjacent medium on TPP is also weakened.

**Figure 6:**
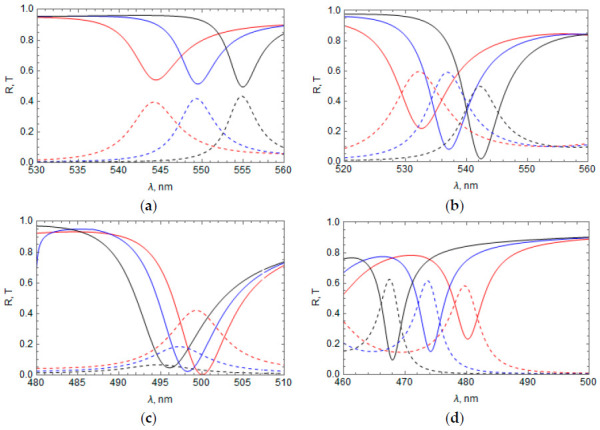
Reflectance and transmittance of the system at different values *n_p_* = 0.15 (red), 0.2 (blue), and 0.25 (black); (**a**) TPP band 1; (**b**) TPP band 2; (**c**) TPP band 3; (**d**) TPP band 4. Thickness of the Ag layer = 35 nm, a = 137.1 nm, $\bar{n}=1.85$.

The TPP wavelength is proportional to the Bragg wavelength $\lambda_{r}=2a\bar{n}$ [3], and there is an obvious shift in the spectral position of the TPP bands with a change in the RF period a and the average refractive index $\bar{n}$. However, the influence of the magnitude of the RF refractive index modulation, $n_{p}$, is not obvious. In Figure 6, we show reflectance and transmittance of the system with fixed a and $\bar{n}$, but with different $n_{p}$ values for the RF refractive index profiles n(z) taken from Table 1.

The RF band gap is proportional to $n_{p}$ and, therefore, broadens with increasing $n_{p}$. This broadening leads to a TPP wavelength shift (see Figure 6), which has an opposite sign for the peaks to the right and left of the RF band gap center (see Figure 2a,b). As a result, the TPP wavelength in the case of bands 1 and 2 increases with increasing $n_{p}$ (Figure 6a,b, respectively), while the TPP wavelength in the case of the bands 3 and 4 decreases with increasing $n_{p}$ (Figure 6c,d, respectively). The magnitude of the reflectance dips and transmittance peaks can change with increasing $n_{p}$ depending on the dip/peak spectral position.

Calculations were also performed for the case when the Ag layer is placed at the bottom of RF. The character of the dependence of reflectance and transmittance on the parameters of the system in this case is the same as in the case of the Ag layer on the top of RF. However, the degree of influence of these parameters decreases significantly with an increasing number of RF periods due to a strong decrease in the electromagnetic field exciting the TPP at the bottom of the RF.

We also compared the results of calculating the reflectance and transmission spectra at $n_{p}=0.2$ using Equations (13)–(16) to results obtained using COMSOL modeling. A difference between these cases is barely noticeable.

**Figure 7:**
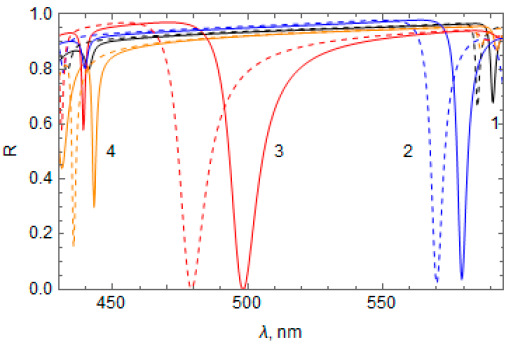
Comparison of the reflectance spectrum of the “Ag layer + RF” calculated using COMSOL software (solid lines) and using Equations (12)–(15) (dashed lines) at $n_{p}=0.5$ TPP bands are numbered according to Table 1: 1 (black), 2 (blue), 3 (red), 4 (orange). $\bar{n}=1.85$, a = 137.1 nm, thickness of the Ag layer = 45 nm.

Lastly, we also calculated the reflectance spectrum of the “Ag layer + RF” system using Equation (13), slightly going beyond the conditions in Equation (6) for applicability of the obtained analytical solution. For this, we set the magnitude of the RF refractive index modulation to be $n_{p}=0.5$. In this case, $\left|\chi_{\pm 1}a\right|\approx 0.43$ and the second condition in Equation (6) is violated. Results of the calculation for all cases of the RF refractive index profile n(z) presented in Table 1 at $n_{p}=0.5$ are shown in Figure 7 (dotted curves). In the same figure, we also show the results of calculating the reflectance spectrum obtained using COMSOL software (solid curves).

It can be seen that, for all considered cases of n(z), the qualitative picture of the spectral distribution of TPP bands obtained using the analytical solution remains the same as in the calculation using COMSOL software. This holds true even when there is a slight violation of the condition in Equation (6), although, in this case, the wavelength of the TPP bands is slightly shifted toward shorter waves, as shown in Figure 7.

## 4. Conclusions

We studied the light reflectance and transmittance of a system composed of a metal (Ag) layer adjacent to a rugate filter having a harmonic refractive index profile. Narrow dips in the reflectance and peaks in the transmittance were obtained, due to the excitation of TPP at the Ag layer–RF interface. We show that parameters of the harmonic profile of the RF refractive index significantly affect the TPP wavelength and magnitude of the TPP dips/peaks. Depending on the profile of the RF refractive index, the spectral position of the TPP can be at any point in the RF band gap. The influence of the metal layer thickness and the external medium refractive index on the position and magnitude of the TPP dips/peaks was also established. It should be noted that the proposed analytical solution describes the spectral position and magnitude of the TPP bands quite well, even when the RF parameters slightly violate the conditions for its derivation.

Lastly, we would like to point out that the potential applications of TPPs on metal with adjacent RF include all areas where TPPs on metal with an adjacent traditional Bragg mirror are applicable, but with the advantages of RF mentioned in Section 1. In particular, the possibility of superimposing several harmonic waves of the refractive index in RF allows for the simultaneous use of several spectral notches, which makes it possible to use TPPs excited in the spectral region of different notches.

We believe that the proposed analytical method for studying plasmonic structures with an RF and the obtained dependences of reflectance and transmittance can be used for designing devices based on Tamm plasmons.

## Figures and Tables

**Table 1:** The RF refractive indices used for numerical calculations.

α	Rugate Filter Refractive Index	Spectral Band Number in Figure 2
−π/2	$n\left(z\right)=\bar{n}-n_{p}\cos\left(2\pi z/a\right)$	1
0	$n\left(z\right)=\bar{n}+n_{p}\sin\left(2\pi z/a\right)$	2
π/2	$n\left(z\right)=\bar{n}+n_{p}\cos\left(2\pi z/a\right)$	3
π	$n\left(z\right)=\bar{n}-n_{p}\sin\left(2\pi z/a\right)$	4

## Data Availability

Data sharing not applicable.
